# Characterization of stemness features and construction of a stemness subtype classifier to predict survival and treatment responses in lung squamous cell carcinoma

**DOI:** 10.1186/s12885-023-10918-y

**Published:** 2023-06-08

**Authors:** Jinzhi Lai, Xinyi Lin, Huangna Zheng, Bilan Xie, Deqiang Fu

**Affiliations:** 1 Department of Oncology, The Second Affiliated Hospital of Fujian Medical University, Quanzhou, 362000 Fujian China; 2 Department of Hematology, The Second Affiliated Hospital of Fujian Medical University, Quanzhou, 362000 Fujian China

**Keywords:** Lung squamous cell carcinoma, Cancer stemness, Treatment response, Prognosis

## Abstract

**Background:**

Cancer stemness has been proven to affect tumorigenesis, metastasis, and drug resistance in various cancers, including lung squamous cell carcinoma (LUSC). We intended to develop a clinically applicable stemness subtype classifier that could assist physicians in predicting patient prognosis and treatment response.

**Methods:**

This study collected RNA-seq data from TCGA and GEO databases to calculate transcriptional stemness indices (mRNAsi) using the one-class logistic regression machine learning algorithm. Unsupervised consensus clustering was conducted to identify a stemness-based classification. Immune infiltration analysis (ESTIMATE and ssGSEA algorithms) methods were used to investigate the immune infiltration status of different subtypes. Tumor Immune Dysfunction and Exclusion (TIDE) and Immunophenotype Score (IPS) were used to evaluate the immunotherapy response. The pRRophetic algorithm was used to estimate the efficiency of chemotherapeutic and targeted agents. Two machine learning algorithms (LASSO and RF) and multivariate logistic regression analysis were performed to construct a novel stemness-related classifier.

**Results:**

We observed that patients in the high-mRNAsi group had a better prognosis than those in the low-mRNAsi group. Next, we identified 190 stemness-related differentially expressed genes (DEGs) that could categorize LUSC patients into two stemness subtypes. Patients in the stemness subtype B group with higher mRNAsi scores exhibited better overall survival (OS) than those in the stemness subtype A group. Immunotherapy prediction demonstrated that stemness subtype A has a better response to immune checkpoint inhibitors (ICIs). Furthermore, the drug response prediction indicated that stemness subtype A had a better response to chemotherapy but was more resistant to epidermal growth factor receptor tyrosine kinase inhibitors (EGFR-TKIs). Finally, we constructed a nine-gene-based classifier to predict patients’ stemness subtype and validated it in independent GEO validation sets. The expression levels of these genes were also validated in clinical tumor specimens.

**Conclusion:**

The stemness-related classifier could serve as a potential prognostic and treatment predictor and assist physicians in selecting effective treatment strategies for patients with LUSC in clinical practice.

**Supplementary Information:**

The online version contains supplementary material available at 10.1186/s12885-023-10918-y.

## Introduction

Cancer stemness is the capacity for self-renewal and differentiation, which leads to tumor metastasis and relapse [1]. In addition, cancer stemness is associated with genomic and proteomic signatures that can modulate malignant biological behaviors and support the initiation, differentiation, and proliferation of tumor cells [2]. Mounting evidence indicates that tumor cells bearing stemness features can differentiate into cancer stem cells (CSCs), which are empowered with increased metastatic capacity and resistance to therapy. Such stem-like cells also exist in various cancers, including lung squamous cell carcinoma (LUSC), and play a critical role in the genetic profile of the tumor microenvironment [3]. Due to the complexity and heterogeneity of the tumor microenvironment, it remains unclear how stemness features regulate stem cell-related biological programs and shape the tumor microenvironment.

LUSC is the top global cause of death with high mortality rates but lacks effective therapeutic strategies [4]. Although the death rate of lung cancer has declined over the past few decades, the average five-year survival rate for LUSC is only between 20% and 30% [5]. Compared with lung adenocarcinoma (LUAD), there are still few effective targeted treatment options for LUSC in the clinic [6]. Unfortunately, LUSC does not respond well to chemotherapy and radiotherapy as well as other types of cancers [7]. In recent years, advances in cancer immunotherapy have extended overall survival (OS) in select non-small cell lung cancer (NSCLC) patients with positive PD-L1 expression. Nevertheless, only a small percentage of LUSC patients show a survival benefit from immunotherapy [8]. Therefore, a subtype classifier based on specific characteristics for survival prediction and therapy response estimation is the first step toward personalized cancer treatment for LUSC patients.

A recent study provided strategies for integrated analysis of cancer stemness features according to the stemness index (mRNAsi), which could classify tumors based on their stemness features and provide predictive biomarkers for treatment response and survival outcome [9]. The stemness index mRNAsi have proven to be associated with the dedifferentiated oncogenic state and infiltrating immune cells of the tumor microenvironment [10]. Furthermore, multiple stemness-related genes have been confirmed to be involved in the prognosis and response to different therapies [11]. However, most existing studies refer to the identification of stemness-related prognostic genes [12, 13]. The relationship among stemness features, tumor heterogeneity, and treatment responsivity in LUSC patients is still unknown. Thus, further integrated analysis of the genetic features of stemness and stemness-related heterogeneity is important for accurate classification and guiding treatment selection for LUSC patients.

In this study, the stemness index (mRNAsi) of LUSC patients was calculated according to mRNA expression data from The Cancer Genome Atlas (TCGA) and GEO databases. Subsequently, LUSC patients were divided into high-mRNAsi and low-mRNAsi groups based on mRNAsi scores, which exhibited distinct survival outcomes and functional annotations. Next, we applied consensus clustering analysis based on stemness-related differentially expressed genes (DEGs) to classify patients into two subtypes with distinct prognoses. Furthermore, bioinformatic analysis were performed to investigate the differences in functional enrichment, immune profiles, and the response to different treatment strategies between these two stemness subtypes. Finally, we constructed a stemness subtype classifier to distinguish these two subtypes and validate the subtype classifier into three independent GEO datasets. Our study provides a clinical practice tool for survival prediction and screening which patients will respond well to immunotherapy, chemotherapy, and targeted therapy.

## Materials and methods

### Data preprocessing and calculation of the stemness index (mRNAsi)

The mRNA-seq data (FPKM), mutation files and corresponding clinicopathological information of 478 LUSC tumor tissues and 50 matched normal samples were collected from the UCSC Xena database (https://xena.ucsc.edu/), and log2 transformation was performed. The mutation annotation format (MAF) of somatic variants was obtained using the “maftools” R package. Three external validation sets, including GSE30219 (n = 61), GSE37745 (n = 66) and GSE73403 (n = 69), were obtained from the GEO database. All three GEO datasets were based on the GPL570 platform, and the “Combat” R package was used to correct for batch effects.

The stemness scores (mRNAsi) of TCGA-LUSC samples were obtained from a previous study [9]. In short, the gene expression data of pluripotent stem cells (PSCs) were collected from the Progenitor Cell Biology Consortium (PCBC) (https://www.synapse.org) database, and the one-class logistic regression (OCLR) machine learning algorithm was applied to calculate the mRNA stemness score (mRNAsi) of each tumor sample [9]. The mRNAsi score ranges from 0 to 1, and a higher mRNAsi score represents strong oncogenic dedifferentiation and stem cell characteristics.

### Analysis of immune cell infiltration and immune status

The “ESTIMATE” algorithm was performed to assess the scores of tumor purity and immune status of each tumor sample based on gene expression profiles [14]. Single-sample gene set enrichment analysis (ssGSEA) was employed to calculate the enrichment score of 13 immune-related terms and 16 immune cells based on 29 immune gene sets by the “GSEAbase” and “GSVA” R packages [15]. We further classified the LUSC patients into high immunity and low immunity groups using hierarchical agglomerative clustering according to the ssGSEA scores. The TIMER [16], QUANTISEQ [17], Microenvironment Cell Populations-counter (MCP-counter) [18] and XCELL [19] algorithms were applied to calculate the abundances of immune cells between the high-mRNAsi and low-mRNAsi groups.

### Differential expression analysis and identification of stemness-related classification

Differentially expressed genes (DEGs) between the high-mRNAsi and low-mRNAsi groups in the TCGA dataset were analyzed using the “limma” R package. A value of |log2-fold change (FC)| >1 and adjusted p value < 0.01 was considered the cutoff criteria for DEG selection.

An unsupervised consensus clustering approach was used to identify stemness-related subtypes of LUSC patients according to the expression of stemness-related genes using the “ConsensusClusterPlus” R package [20]. The consensus clustering analysis was performed with 1000 iterations, and each iteration resampled 80% of the data. Cumulative distribution function (CDF) curves and consensus matrix (CM) plots were used to determine the optimal number of categories (k-means clustering).

### Functional enrichment and gene set variation analysis (GSVA)

Kyoto Encyclopedia of Genes and Genomes (KEGG) and Gene Ontology (GO) enrichment analyses were applied to identify the most impactful pathways and biological functions of these stemness-related DEGs by the “clusterProfiler” and “org.Hs.e.g.db” R packages. A p value < 0.05 and q value < 0.05 were considered significant in this section.

Gene set variation analysis (GSVA) is an unsupervised tool that can be applied to evaluate the variation in the gene set enrichment of each sample [21]. Here, we conducted GSVA to evaluate the enrichment of functional pathway activity between two stemness subtypes by the “GSVA” and “GSEABase” R packages. Differences with an adjusted p < 0.05 were considered statistically significant.

### Prediction of immunotherapy and drug sensitivity analysis

Tumor Immune Dysfunction and Exclusion (TIDE) (http://tide.dfci.harvard.edu/) is a computational method to evaluate responsiveness to immunotherapy according to the T-cell dysfunction score and exclusion score based on gene expression profiles [22]. The TIDE algorithm was used to predict patients’ response to immune checkpoint inhibitor (ICI) therapy between two stemness subtypes. The immunophenotype score (IPS) is a machine learning scoring model ranging from 0 to 10 based on the expression of representative gene sets (https://tcia.at/home), which was applied for the prediction of patients’ response to ICIs [23].

The “pRRophetic” R package was used to predict the clinical efficacy of the patients’ response to chemotherapeutic and targeted therapy. The drug response was evaluated by the half maximal inhibitory concentration (IC50) of each LUSC patient according to the Genomics of Drug Sensitivity in Cancer (GDSC) database [24]. The lower the IC50 value is, the more sensitive cells are to specific chemotherapeutic and targeted agents.

### Development and validation of the stemness-related classifier

All 478 LUSC patients from the TCGA dataset were randomly split into a training set (n = 335) and an internal testing set (n = 143) at a 7:3 ratio. The differentially expressed genes (DEGs) between the high mRNAsi and low mRNAsi groups were considered stemness-related DEGs. Next, the least absolute shrinkage and selection operator (LASSO) and random forest (RF) were applied to further reduce and screen the most critical stemness-related features by the “glmnet” and “randomForest” R packages [25–27]. The stemness-related DEGs were used as the entry parameter, and the stemness cluster was used as the outcome (binary variables, 0 or 1) [28]. The intersection of genes between the LASSO and RF analyses were regarded as the most relevant stemness-related genes. Subsequently, multivariate logistic regression analysis was conducted on these hub stemness-related genes to develop the classification model. The formula of the stemness-related classifier was:

1 $$\displaylines{Stemness{\text{ }}subtype{\text{ }}classifier = \cr \sum\limits_{i = 1}^n {Coe{f_i} * {\chi _i}} + Intercept \cr}$$

$Coe{f_i}$ and ${\chi _i}$ represent the coefficient index and the expression value of genes, respectively. The classifier was normalized in the range [0 to 1]. The performance of the stemness subtype classifier was validated by the time-dependent receiver operating characteristic (ROC) curve to choose the optimal cutoff value. In addition, the stemness subtype classifier was validated in an internal testing set and external validation sets.

### Validation of hub genes in clinical samples by quantitative real-time PCR (qRT-PCR)

We obtained 10 tumor samples and 10 paired adjacent normal samples from LUSC patients at The Second Affiliated Hospital of Fujian Medical University. Our research was approved by the Ethics Committee of the hospital.

The total RNA of clinical samples was isolated by TRIzol reagent (Invitrogen, USA). One milliliter of TRIzol per 50–100 mg of LUSC tissue was added to the sample and homogenized by a homogenizer. Total RNA was extracted from tissues using TRIzol reagent according to the manufacturer’s protocol and was reverse transcribed to cDNA with the PrimeScript 1st Strand cDNA Synthesis Kit (TaKaRa, Japan). Quantitative RT-PCR was conducted with the SYBR-Green method using SYBR Premix Ex Taq (TaKaRa, Japan). The sequences of the PCR primers used in this study were synthesized by Sangon Biotech (Shanghai, China) and are listed in Table S1. GAPDH levels served as the internal quantity control. Relative mRNA expression levels were calculated by the delta-delta-Ct method.

### Statistical analysis

The statistical analyses were performed using R software version 4.1.3. The student’s t-test was utilized to compare normally distributed data between two groups, while the Chi-square test was employed to compare categorical and pairwise features across different groups. The Mann-Whitney U test was used to determine statistically significant differences between two groups, while the Kruskal-Wallis test was employed to evaluate statistically significant differences among multiple independent groups. Pearson’s correlation test was used to assess correlations between normally distributed variables, and Spearman’s correlation test was utilized to evaluate correlations between non-normally distributed variables. The Kaplan-Meier method and log-rank test were applied to analyze survival differences between two or more groups. All tests were two-sided, and statistical significance was defined as a p-value less than 0.05, unless otherwise specified.

## Results

### A high stemness index was associated with better patient prognosis in LUSC

**Fig. 1:**
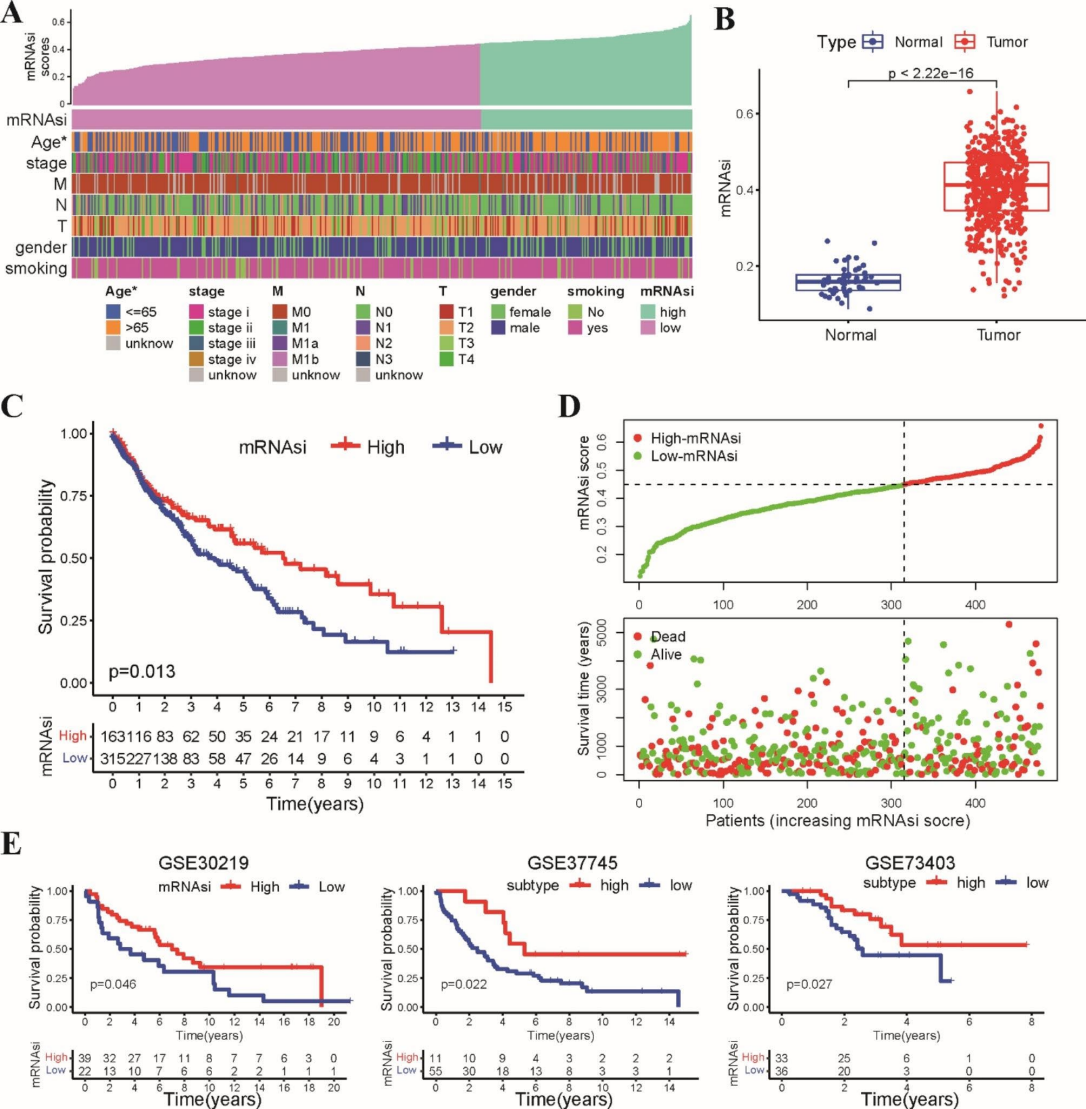
High stemness index was associated with the better patients’ prognosis in LUSC. **A** An overview of the relationship between mRNAsi scores and clinicalpathological features of LUSC patients. **B** The scatter plot showed that mRNAsi scores in tumor tissues were higher than that in normal tissues in LUSC. **C** Kaplan–Meier survival curve showed that patients in high-mRNAsi group had better OS than those in low-mRNAsi group. **D** The distribution of mRNAsi scores and survival status in TCGA dataset. **E** Kaplan-Meier curves indicated that patients of high-mRNAsi group exhibited longer OS in three GEO independent datasets

The workflow of our study is shown in Figure S1. First, the correlation between the stemness index and clinicopathological characteristics was analyzed based on the mRNAsi scores of LUSC patients from the TCGA dataset. Patients aged < 65 years had higher mRNAsi scores than patients aged ≥ 65 years (Fig. 1A). There was no significant difference among patients of different gender, T stages, N stages and clinical stages (Fig. S2A). The mRNAsi scores in normal samples were lower than those in tumor tissues (Fig. 1B). A positive correlation between mRNAsi scores and TMB was observed (Fig. S2B). The waterfall plots displayed the 20 frequently mutated genes in these two groups (Fig. S2C). Next, we systematically examined the correlation between the stemness index and patient prognosis in LUSC. We found that patients in the high-mRNAsi group exhibited better OS than those in the low-mRNAsi group (Fig. 1C). Patients in the high-mRNAsi group presented a lower probability of death than those in the low-mRNAsi group with an increase in the stemness index (Fig. 1D). We further validated the association between the stemness index and prognosis in three GEO independent datasets (GSE30219, GSE37745 and GSE73403). As expected, patients in the high-mRNAsi group had longer OS than those in the low-mRNAsi group (Fig. 1E). Altogether, these results indicated that the increase in mRNAsi scores was closely associated with a better prognosis in LUSC.

### Correlation between the stemness index and immune landscape in the tumor microenvironment

**Fig. 2:**
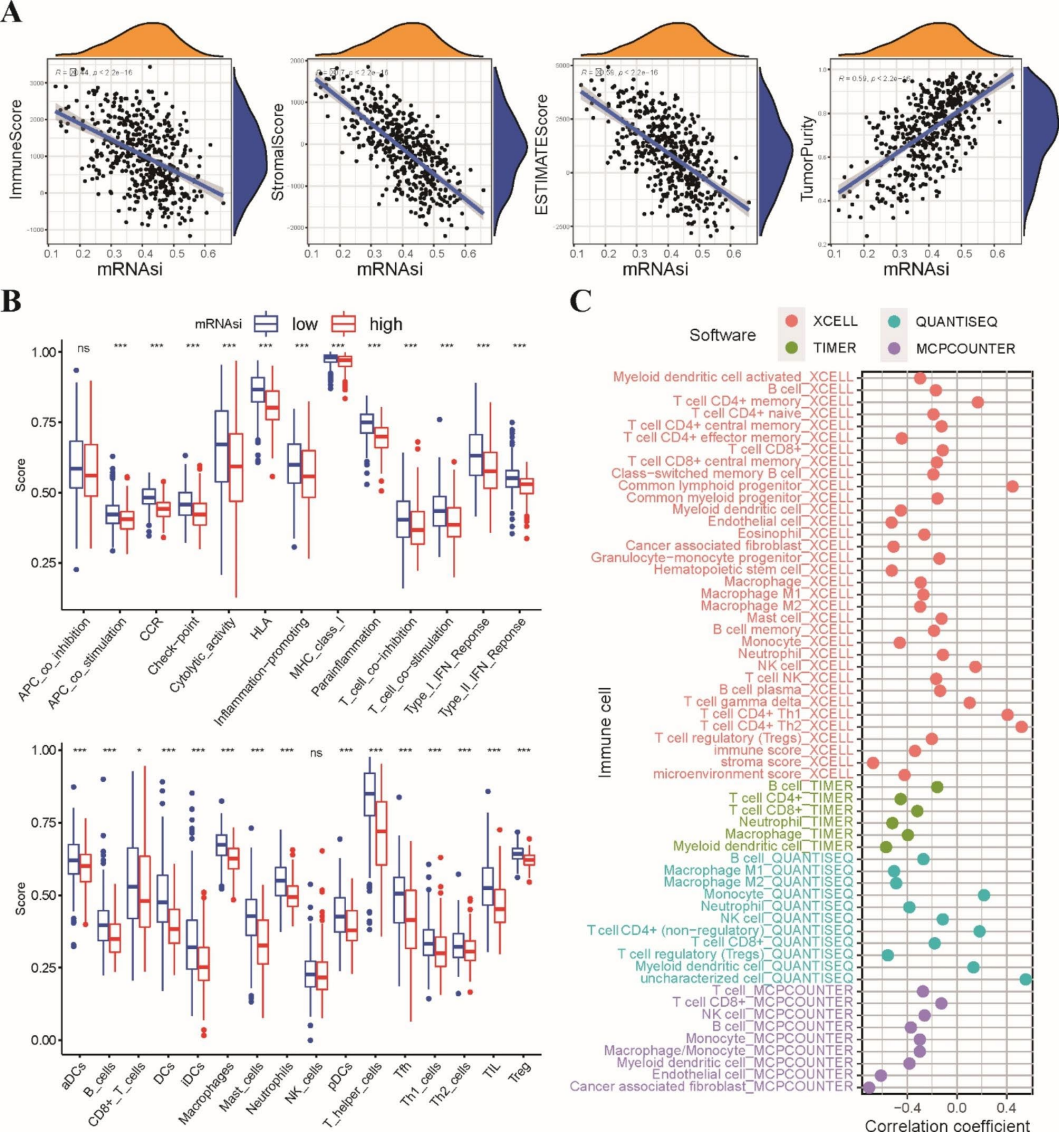
Correlation between stemness index and immune landscape of tumor microenvironment in LUSC. **A** Correlation analysis of immune score, stromal score, ESTIMATE score and tumor purity with mRNAs scores. **B** Differences in the enrichment scores of representative immune-related pathways and immune-infiltrating cells between the low-mRNAsi and high-mRNAsi groups. **C** Correlation analysis of the mRNAsi scores and immune-infiltrating cells in LUSC. * p < 0.05, ** p < 0.01, *** p < 0.001

An increasing number of studies have reported that cancer stemness is closely associated with immune cell infiltration [29]. We first investigated the relationship between the stemness index and tumor immune status in the TCGA dataset by the ESTIMATE algorithm. Correlation analysis demonstrated that the mRNAsi score was positively correlated with tumor purity but negatively correlated with the immune score, stromal score, and ESTIMATE score (Fig. 2A). These results suggested that the levels of tumor-infiltrating immune cells decreased with increasing mRNAsi score in LUSC patients. Next, the ssGSEA algorithm based on 29 immune gene sets was used to quantify the immune-related pathways and relative abundance of immune infiltrating cell subpopulations. Consistent with the ESTIMATE analysis, we observed that the low-mRNAsi group was enriched in most immune-related pathways, including T-cell costimulation/inhibition, APC costimulation/inhibition and I/II-IFN response, and most types of immune cells were elevated in the low-mRNAsi group (Fig. 2B). In addition, we utilized multiple algorithms, including TIMER, QUANTISEQ, MCP-COUNTER and XCELL, to estimate the relationship between immune cells and the mRNAsi score. We found that the mRNAsi score was negatively correlated with the main types of immune cells (Fig. 2C). Taken together, these data indicated that the stemness index was negatively correlated with low tumor immune status in LUSC.

### Identifying stemness-related DEGs and functional enrichment analysis

**Fig. 3:**
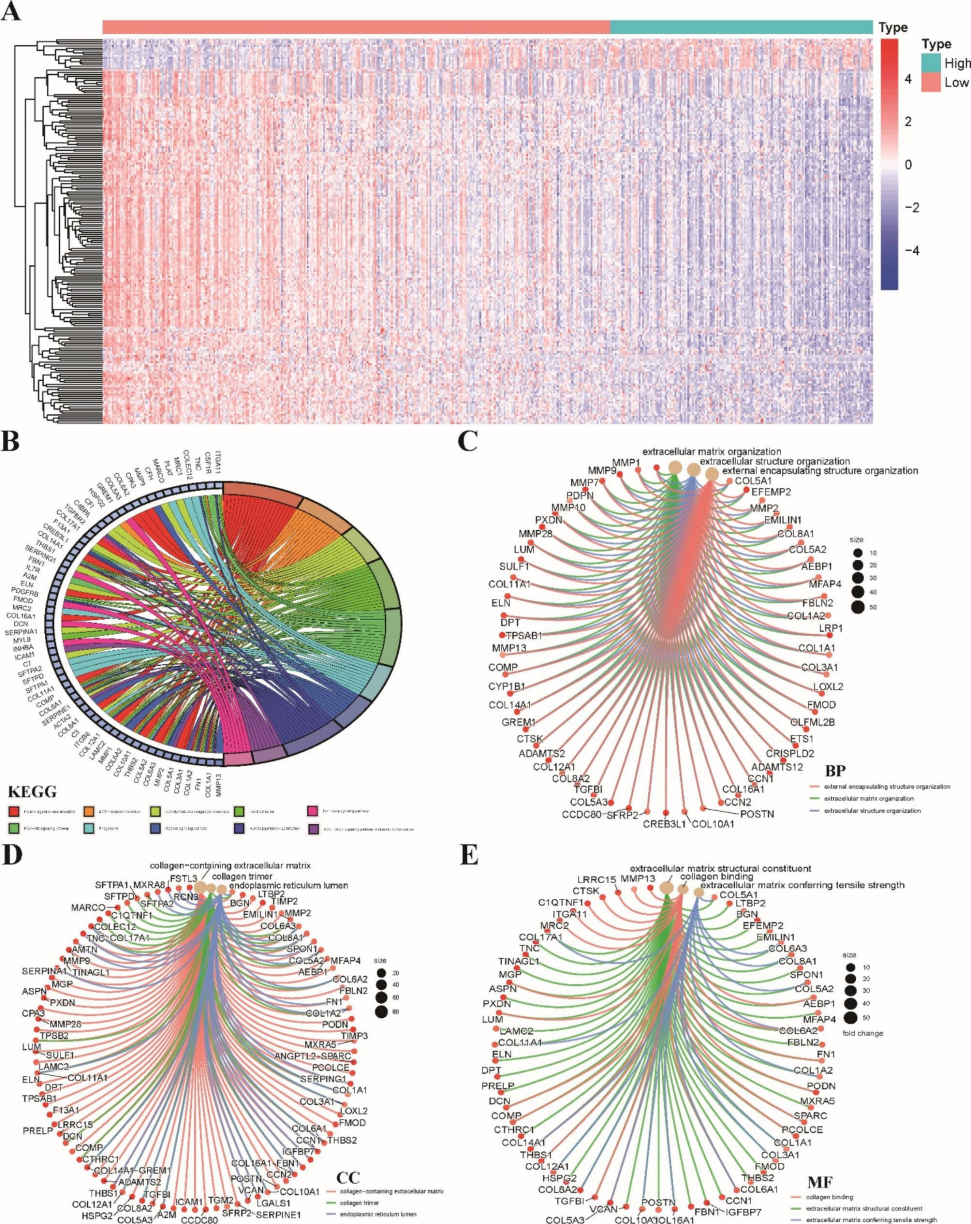
Identifying stemness-related DEGs and functional enrichment analysis. **A** Heatmap demonstrated the expression levels of 190 DEGs between the low-mRNAsi and high-mRNAsi groups. **B** KEGG functional enrichment analyses of 190 DEGs. The GO enrichment analyses of 190 DEGs including biological processes **(C)**, cellular components **(D)** and molecular functions **(E)** in LUSC.

Because the stemness index was closely associated with OS and the tumor immune microenvironment status of LUSC, we conducted further investigations into the difference between the high mRNAsi and low mRNAsi groups. The GSVA enrichment analysis revealed a significant enrichment of pathways related to DNA damage repair in the high mRNAsi group. These pathways included homologous recombination, mismatch repair and nucleotide excision repair pathways (Fig. S3A). A total of 190 stemness-related DEGs were screened based on the selected thresholds (|log2-fold change (FC)| >1 and adjusted p value < 0.01), including 15 upregulated genes and 175 downregulated genes (Fig. 3A). Next, functional enrichment analysis was used to evaluate the biological functions and signaling pathways of these 190 stemness-related DEGs. KEGG pathway analysis showed that these DEGs were enriched in pathways related to the tumor microenvironment, including protein digestion and absorption, ECM-receptor interaction and complement and coagulation cascade pathways. Several classical cancer-related pathways also emerged, including the PI3K-AKT signaling and TGF-beta signaling pathways (Fig. 3B). The GO enrichment analysis indicated that these DEGs participated in (BP terms) external encapsulating structure organization (Fig. 3C), (CC terms) collagen containing extracellular matrix (Fig. 3D) and (MF terms) extracellular matrix structural constituent (Fig. 3E).

### Identification of two stemness subtypes based on stemness-related DEGs

**Fig. 4:**
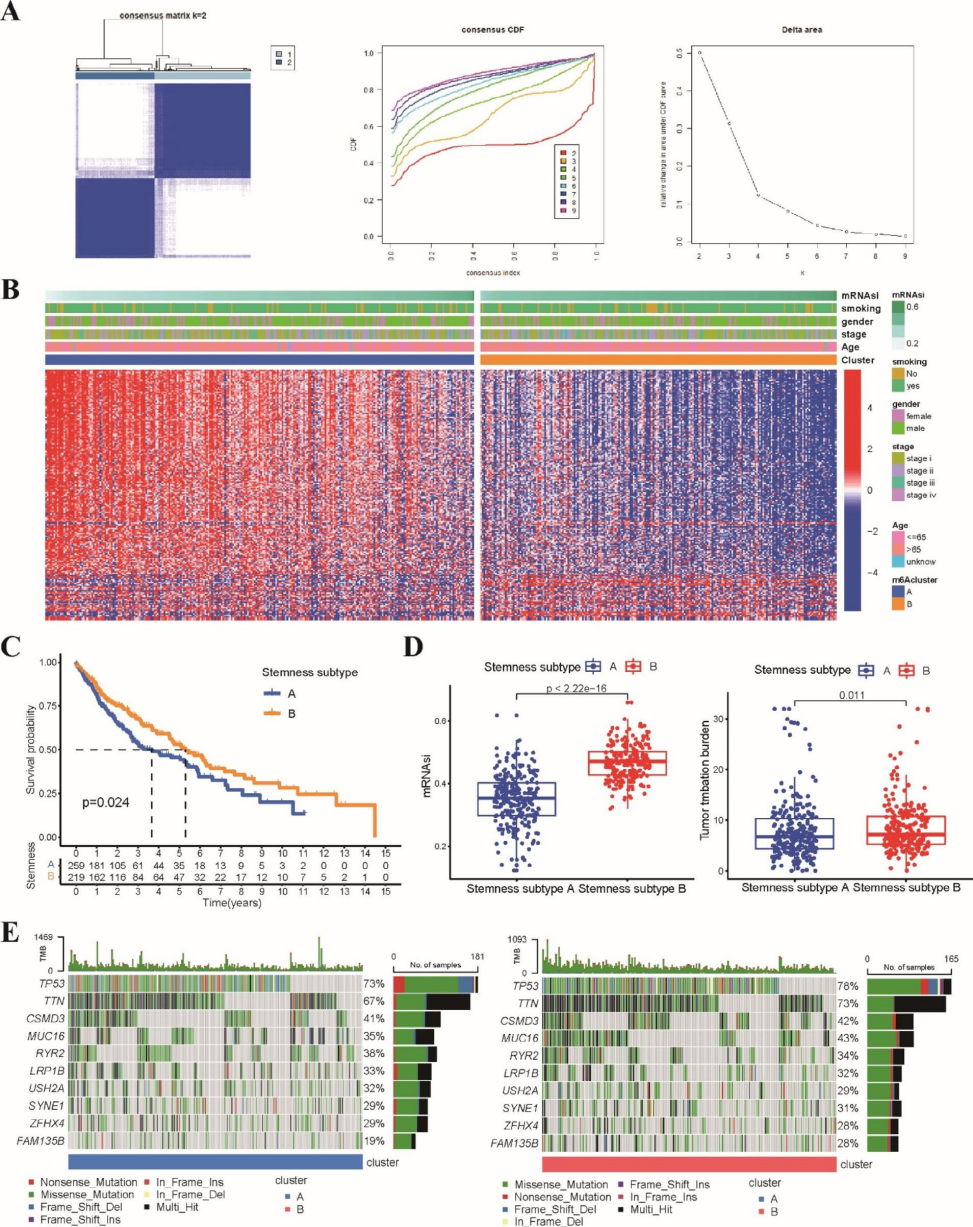
Identification of two stemness subtypes based on stemness-related DEGs. **A** Consensus clustering of LUSC patients based on 190 stemness-related DEGs. CDF curves of the consensus score and the relative change in area under the CDF curve from k = 2 to 9. **B** The heatmap of the expression of DEGs and the clinicopathological characteristics between stemness subtype A and B groups. **C** Kaplan–Meier analysis exhibited patients in stemness subtype B had a better prognosis compared to stemness subtype A. **D** Comparisons of mRNAsi scores and TMB values between two stemness subtypes. **E** Waterfall plots displayed the top frequently mutated genes between two stemness subtypes

To further analyze the heterogeneity of stemness characteristics, an unsupervised consensus clustering method was applied to identify a new molecular cluster of LUSC patients based on 190 stemness-related DEGs. According to the consensus heatmap and the relative change in the area under the CDF curve, k = 2 was the optimal value for clustering (Fig. 4A). All LUSC patients were classified into 2 stemness subtypes, including stemness subtype A (259 patients, 54.2%), which tended to have a lower mRNAsi score, and stemness subtype B (219 patients, 45.8%), which had a higher mRNAsi score (Fig. 4B). The Kaplan‒Meier curve demonstrated that LUSC patients with stemness subtype B exhibited a longer OS than patients with stemness subtype A (Fig. 4C). The mRNAsi score and TMB value in the stemness subtype A group were lower than those in the stemness subtype B group (Fig. 4D). The mutation statuses of TP53, TTN and CSMD3 were statistically significant between the two subtypes, and waterfall plots displayed high-frequency mutations of different genes between the two stemness subtypes (Fig. 4E). Furthermore, patients in stemness subtype A were older than those in stemness subtype B. No significant difference was found in gender, TNM stage, clinical stage or smoking status between the two stemness subtypes (Fig. S3B).

### Two stemness subtypes possessed different functional annotations and immune microenvironments

**Fig. 5:**
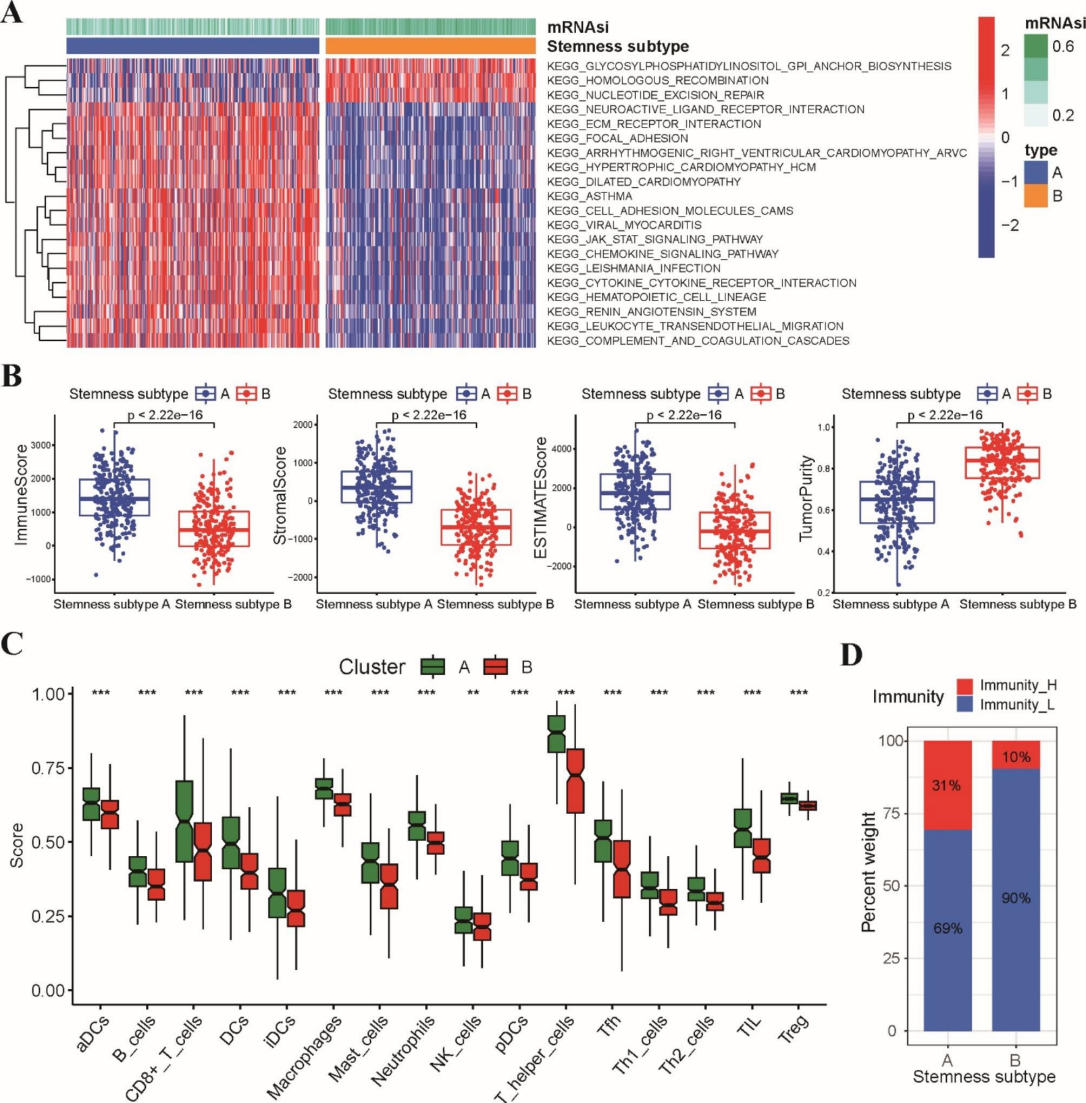
Two stemness subtypes possessed different functional annotations and immune microenvironment. **A** Heatmap of GSVA analysis demonstrated the top 20 significantly enriched molecular pathways between stemness subtype A and B groups. **B** Comparisons of immune score, stromal score, ESTIMATE score and tumor purity between stemness subtype A and B groups. **C** Comparisons of the proportions of immune infiltration cells between stemness subtype A and B groups. **D** Stacked histogram showing the proportions of immunity classification in stemness subtype A and B groups. * p < 0.05, ** p < 0.01, *** p < 0.001

To elucidate the potential differences in molecular pathways related to the two stemness subtypes, GSVA enrichment analysis was implemented to assess the cluster-specific signaling pathways. We found that pathways related to DNA damage repair, such as homologous recombination and nucleotide excision repair pathways, were enriched in the stemness subtype B group. However, the stemness subtype A group mainly correlated with immune-related pathways, including the chemokine signaling pathway, cytokine‒cytokine receptor interaction and leucocyte transendothelial migration pathway, and pathways related to the TME, such as ECM-receptor interaction, cell adhesion molecules and focal adhesion pathways (Fig. 5A). The results demonstrated that genomic differences might generate distinct immune infiltration statuses between the two stemness subtypes.

Subsequently, we performed ESTIMATE and ssGSEA analyses to investigate the immune infiltration status between the two stemness subtypes. The results of ESTIMATE analysis showed that ESTIMATE scores, immune scores and stromal scores were higher in stemness subtype A, while a higher tumor purity score was observed in stemness subtype B (Fig. 5B). ssGSEA revealed that almost all immune cell types were more abundant in stemness subtype A (Fig. 5C). Furthermore, the aforementioned immune classification showed that stemness subtype A consisted of most of the high-immunity patients (Fig. 5D). These findings suggested that the stemness subtype A group had a relatively higher immune infiltration status than the stemness subtype B group.

### Different stemness subtypes possess distinct immunotherapy responses and drug sensitivities

**Fig. 6:**
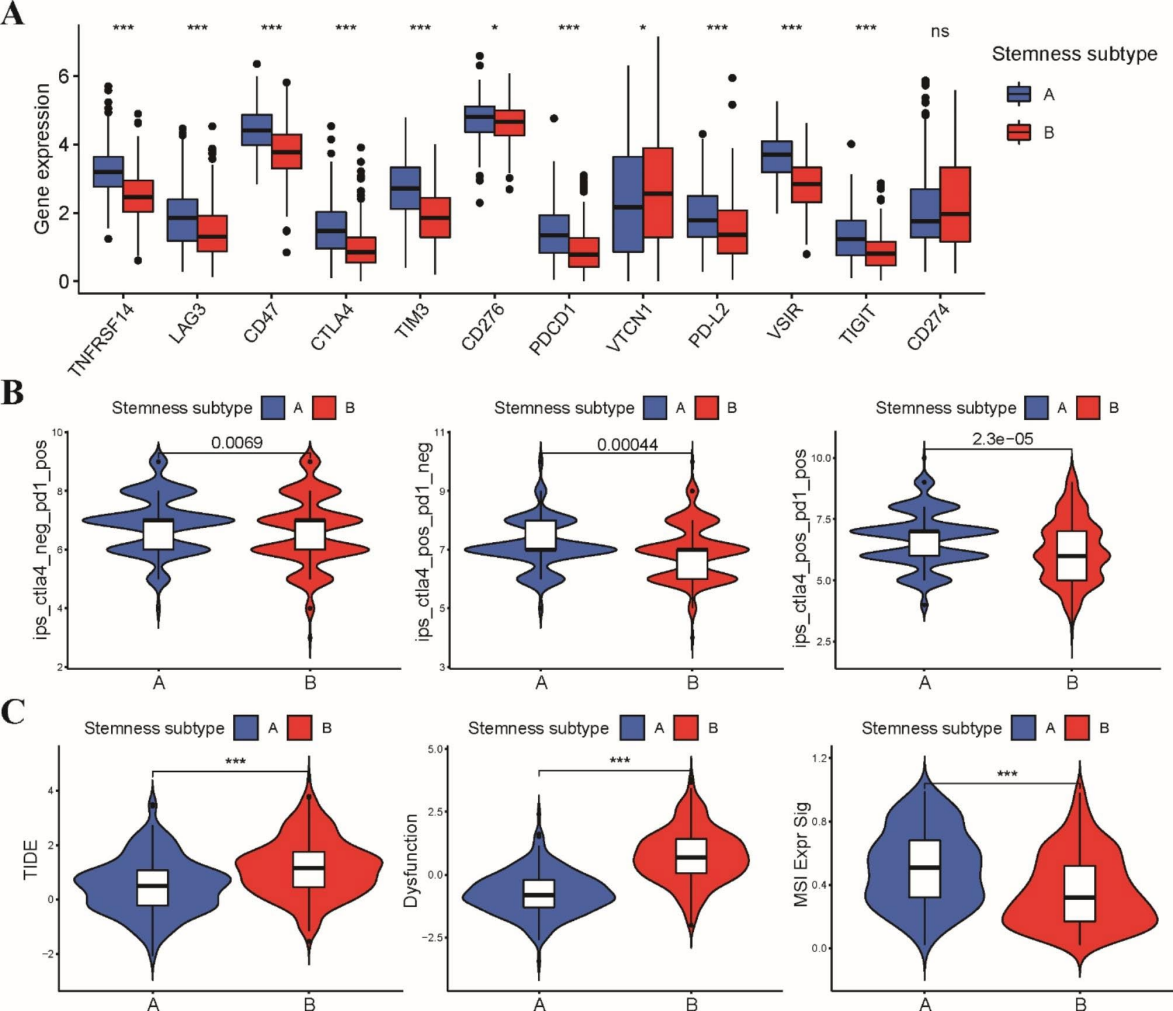
Evaluation of immunotherapy sensitivity between these two stemness subtypes in TCGA-LUSC dataset. **A** Expression profile of the expression of representative immune checkpoint genes between stemness subtype A and B groups. **B** The relative probabilities to respond to PD-1 and CTLA-4 inhibitors in stemness subtype A and B groups. **C** The TIDE, T cell dysfunction and MSI scores in stemness subtype A and B groups. * p < 0.05, *** p < 0.001

Herein, we further explored the expression profile of multiple immune checkpoint genes between the two stemness subtypes. The expression of most of the immune checkpoint genes, including PD1, PD-L1, CTLA-4, TIM3 and Lag3, was significantly higher in stemness subtype A (Fig. 6A), illustrating that patients with stemness subtype A might be more sensitive to ICIs. Subsequently, the IPS and TIDE algorithms were applied to estimate immunotherapy sensitivity in LUSC patients. As expected, patients with stemness subtype A had a higher IPS than patients with stemness subtype B, suggesting a positive response to both PD-1 and CTLA-4 inhibitors (Fig. 6B). The TIDE results also showed that patients in the stemness subtype A group had a lower TIDE score but higher MSI and T-cell dysfunction scores than those in the stemness subtype B group (Fig. 6C). These data indicated that patients with stemness subtype A were more likely to benefit from ICI administration.

**Fig. 7:**
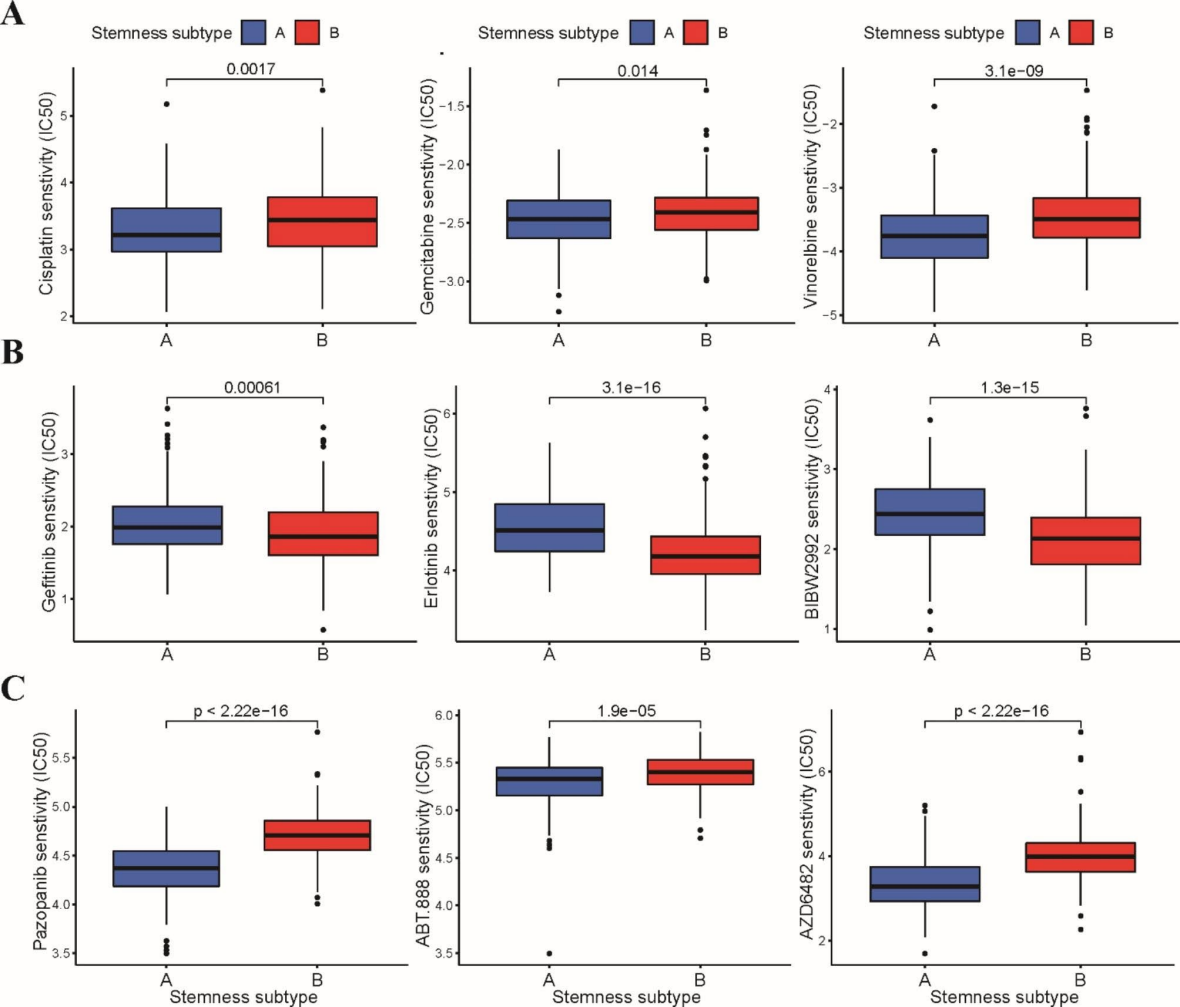
Evaluation of drug sensitivity between these two stemness subtypes in TCGA-LUSC dataset. **A** Differences between stemness subtype A and B groups in response to chemotherapy drugs including cisplatin, gemcitabine and vinorelbine. **B** The IC50 values of three EGFR-TKIs, erlotinib, gefitinib and afatinib between stemness subtype A and B groups. **C** The IC50 values of VEGFR, PARP1 and PI3K inhibitors between stemness subtype A and B groups

Additionally, we further investigated whether these two stemness subtypes might respond differently to chemotherapeutics and targeted drugs. According to the pRRophetic algorithm, we found that the standard first-line chemotherapy drugs, including cisplatin, gemcitabine and vinorelbine, had lower IC50 values in stemness subtype A than in subtype B, indicating a higher chemotherapy sensitivity in patients with stemness subtype A (Fig. 7A). Epidermal growth factor receptor tyrosine kinase inhibitors (EGFR-TKIs) are an effective treatment for EGFR-mutant non-small cell lung cancer (NSCLC). We estimated the IC50 of EGFR-TKIs, which showed that the IC50 values of both first-generation and second-generation EGFR-TKIs were significantly lower in patients with stemness subtype B (Fig. 7B). However, the IC50 values of other targeted inhibitors, such as VEGFR, PARP1 and PI3K inhibitors, were lower in stemness subtype A (Fig. 7C).

### Establishment and validation of the stemness subtype classifier

**Fig. 8:**
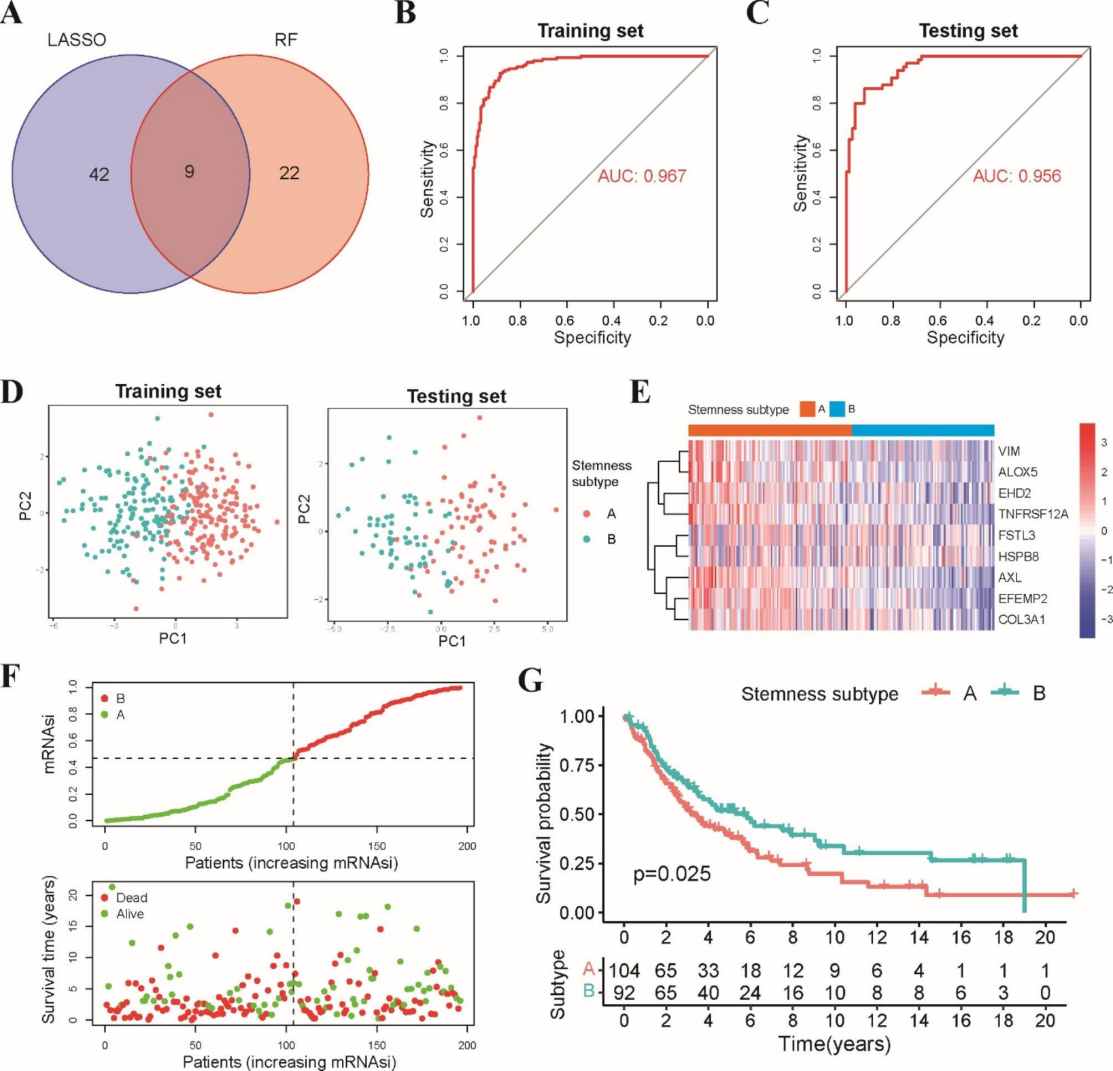
Establishment and validation of stemness subtype classifier. **A** Venn diagram showed that nine stemness-related genes were shared by LASSO and RF regression analysis. **B** ROC curve illustrated that stemness subtype classifier was reliable in distinguishing two subtypes in training set and (**C)** testing set. **D** PCA plot based on the stemness subtype classifier in the training set and testing set. **E** The heatmap of nine stemness-related genes in GEO validation set. **F** Distribution of scores and survival status in stemness subtype A and B in GEO validation set. **G** Kaplan-Meier curve revealed that patients in stemness subtype B group exhibited better prognosis than those in stemness subtype A group in GEO validation set

Finally, we tried to develop a clinically applicable stemness subtype classifier that could easily discriminate the stemness subtype of LUSC patients. LUSC patients from the TCGA dataset were randomly split into a training set (n = 335) and a testing set (n = 143) at a 7:3 ratio. LASSO and RF analyses were applied to select the most hub genes associated with the stemness subtypes based on the expression of 190 stemness-related DEGs (Fig. S4A-B). A total of 51 and 31 stemness-related DEGs were selected by these two machine learning algorithms. After the intersection, nine hub genes were shared by these two feature selection methods (Fig. 8A). Next, multivariate logistic regression analysis was employed to develop a stemness subtype classifier by incorporating these nine genes. The formula of the stemness subtype classifier was 25.426 + (-0.473 × expression of AXL) + (-0.667 × expression of EFEMP2) + (-1.088× expression of VIM) + (-0.1227 × expression of EHD2) + (-0.397 × expression of COL3A1) + (-0.379 × expression of FSTL3) + (-0.557 × expression of ALOX5) + (-0.552 × expression of TNFRSF12A) + (-0.229 × expression of HSPB8). The optimal cutoff score for the classifier to discriminate these two subtypes was 0.49, indicating that patients with scores < 0.49 were assigned to stemness subtype A, while the others were assigned to stemness subtype B. ROC curves illustrated that the stemness subtype classifier was very reliable in distinguishing these two stemness subtypes with an AUC of 0.967 (Fig. 8B). The sensitivity, specificity and accuracy were 88.16%, 91.26% and 89.9%, respectively, in the training set. Furthermore, the stemness subtype classifier also showed good performance in the classification of these two stemness subtypes in the testing set, with an AUC of 0.956, and the sensitivity, specificity, and accuracy were 86.15%, 91.03% and 88.9%, respectively (Fig. 8C). Principal component analysis (PCA) validated that the stemness subtype classifier could split LUSC patients into two stemness subtypes in both the training and testing sets (Fig. 8D).

To further validate the significance of our stemness subtype classifier, three GEO datasets (GSE30219, GSE37745 and GSE73403) were enrolled as the external validation set. We calculated the score of each patient based on the same formula and then classified these patients into two subgroups according to the cutoff score of the classifier (Fig. 8E-F). Patients in three GEO validation datasets were classified into 2 stemness subtypes, including 104 patients in stemness subtype A (53.1%) and 96 patients in stemness subtype B (46.9%). Similar to TCGA results, the Kaplan–Meier curve revealed that patients with stemness subtype B had a better OS than those with stemness subtype A (Fig. 8G). Taken together, these results indicated that the stemness subtype classifier was a reliable tool in discriminating the stemness subtypes and could serve as an independent predictor for the prognosis of LUSC patients.

### Validation of the expression of bub genes using qRT-PCR

**Fig. 9:**
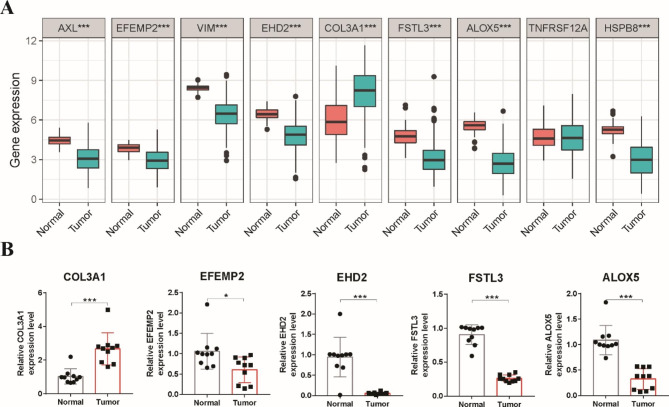
Validation of the expression of hub genes using qRT-PCR. **A** The expression levels of nine hub genes in tumor and normal tissues in TCGA dataset. **B** The expression levels of COL3A1, EFEMP2, EHD2, FSTL3 and ALOX5 in 10 paired LUSC tumor and adjacent normal tissues were quantified by qRT-PCR. * p < 0.05, ** p < 0.01, *** p < 0.001

To validate the results of the stemness subtype classifier, we used qRT‒PCR to validate the expression levels of these hub genes in clinical specimens. As shown in Fig. 9A, seven hub genes (AXL, EFEMP2, VIM, EHD2, FSTL3, ALOX5, HSPB8) were downregulated in tumor samples, while COL3A1 was upregulated in tumor samples in the TCGA dataset. We further validated these 8 genes in 10 paired LUSC tumor and adjacent normal tissues using qRT-PCR. We found that the expression levels of EFEMP2, EHD2, FSTL3 and ALOX5 were decreased in tumor tissues, whereas COL3A1 was significantly increased in tumor samples (Fig. 9B). We did not detect the expression of AXL, VIM or HSPB8. These laboratory data were consistent with the results of previous bioinformatics results, which partially supported the credibility of the classifier.

## Discussion

Due to tumor intrinsic heterogeneity and complex genomics, new cancer treatments for LUSCs have been challenging in recent years [3]. It is urgent to determine the interplay of oncogenic pathways and develop new therapies available for LUSC patients. Cancer stemness is associated with particular oncogenic pathways that can modulate transcriptional networks and support cancer cell growth, proliferation and metastasis [30]. Furthermore, cancer stemness can effectively quantify the level of oncogenic differentiation in tumor tissue via the mRNA expression-based stemness index (mRNAsi) [9]. Recent studies revealed that cancer stemness could affect the treatment response and clinical outcome in different types of cancer, including LUSC [31, 32]. Our study performed an in-depth analysis of the correlation between cancer stemness and the efficacy of immunotherapy, chemotherapy, and targeted therapy in patients with LUSC. We presented an approach to discriminate tumor subtypes with distinct treatment responses and prognoses according to the stemness index and validated this approach through its application to multiple independent datasets.

Here, we calculated the stemness index (mRNAsi) of LUSC patients from the TCGA and GEO databases via the OCLR algorithm. The mRNAsi was low in normal samples but high in tumor samples, which was consistent with the point that tumor progression involved the acquisition of oncogenic dedifferentiation and stemness features. After performing an integrated analysis of the connection of mRNAsi with the survival outcome, we observed that the stemness index was positively associated with OS, while no significant difference in PFS was found between the low mRNAsi group and the high mRNAsi group, indicating that high mRNAsi was an indicator of favorable OS for LUSC patients. Interestingly, a negative association between the stemness score and survival was reported in some cancers, such as pancreatic cancer and liver cancer [33, 34]. The disparate results indicated that the association between the mRNAsi score and survival outcome across different tumor types is complex and likely involves multiple factors, cancer stemness may be linked to the origins of malignant cells and the heterogeneity of tumors in certain types of cancer. In the case of LUSC, several factors may contribute to this finding. Firstly, we observed that patients in the high mRNAsi group tended to be younger than those in the low mRNAsi group. Secondly, our gene set variation analysis (GSVA) revealed a significant enrichment of pathways related to DNA damage repair in the high mRNAsi group, including homologous recombination, mismatch repair, and nucleotide excision repair pathways. It is widely acknowledged that the DNA repair capacity of tumors is an important prognostic factor in cancer patients. Therefore, further research is needed to fully elucidate the underlying mechanisms behind the observed association between mRNAsi scores and prognosis in LUSC.

Mounting evidence suggests that stemness is associated with immune microenvironment variables and the antitumor immune response [10]. Our results showed that the mRNAsi score was negatively correlated with most tumor-infiltrating immune cells, and the results were truly unexpected. However, several studies have reported that there is a negative association between cancer stemness and immune infiltration [29, 35]. The tumor immune microenvironment is diverse and complex in terms of immune status, and complex interactions among tumors, immune cells and their microenvironment exist throughout the initiation and development of tumors. Tumor-infiltrating immune cells may perform either protumorigenic or antitumor roles, which could shape their microenvironment and affect tumor development and the prognosis of patients [36]. Our results demonstrated that most immune cells were increased in the low mRNAsi group, including immunosuppressive cells such as regulatory T cells (Tregs) macrophages and tumor-associated macrophages, which were associated with poor prognosis. Another possible explanation for the high CD8 T-cell abundance in the low-mRNAsi group with poor prognosis may be that several immunosuppressive molecules, including PD-1, TIM3, and LAG3, were also higher in the low-mRNAsi group. High PD-1 and TIM3 expression on CD8 T cells was associated with exhaustion status, which may contribute to the poor prognosis of patients with lung cancer [37, 38]. In addition, tumor-infiltrating immune cells may vary in their activation status under different stimulators.

Immunotherapy, such as ICIs, has revolutionized the treatment options for LUSC owing to its durable response but manageable side effects and is currently approved as the first-line treatment for patients with advanced LUSC [4, 8]. However, a large proportion of LUSC patients do not respond to cancer immunotherapy. Based on the above issue of clinical efficacy in immunotherapy, our study constructed a novel LUSC classification according to tumor stemness. LUSC patients were divided into stemness subtype A and stemness subtype B based on the expression of stemness-related DEGs. We observed that patients in stemness subtype A with lower mRNAsi scores responded better to immunotherapy than those in stemness subtype B. Various factors may influence the response to immunotherapy in lung cancer. Taking ICIs as an example, PD-L1 expression, tumor-infiltrating lymphocytes, tumor mutation burden (TMB) and mismatch repair deficiency status were all able to affect the efficacy of ICIs [39, 40]. Our results showed that stemness subtype A tended to manifest as increased expression of immune coinhibitory/costimulatory genes, including PD-L1, enrichment of immune-related pathways and high immune status, which could explain why patients with stemness subtype A have a better response to immunotherapy.

At present, platinum-doublet chemotherapy is still the standard of treatment for patients with unresectable LUSC [41, 42]. We found that patients with stemness subtype A showed higher sensitivity to first-line chemotherapeutic drugs, including cisplatin, gemcitabine and vinorelbine. Our results were consistent with previous reports that a higher stemness index was correlated with chemoresistance due to its self-renewal ability and drug-efflux ability [43]. Furthermore, DNA damage repair pathways are important determinants of sensitivity to chemotherapeutic agents [44]. GSVA showed that DNA damage repair pathways, including homologous recombination and nucleotide excision repair pathways, were enriched in stemness subtype B, which may contribute to chemoresistance in the stemness subtype B group. Targeted therapies with EGFR-TKIs have shown very limited clinical benefits in treating LUSC patients [45]. Our study showed that patients with stemness subtype B were more sensitive to EGFR-TKIs and resistant to VEGFR, PARP1 and PI3K inhibitors. The underlying cancer stemness ability is dependent on multiple molecular targets, including signaling pathways, the tumor microenvironment and stem cell differentiation. These molecular targets may be involved in the efficiency of tumor chemotherapy and targeted therapy, indicating that the combination of chemotherapy, targeted therapy or immunotherapy may provide more efficient management to eliminate cancer stemness in LUSC patients.

To apply our results in clinical practice, we developed a clinically applicable classifier that could easily discriminate the stemness subtype of LUSC patients based on 190 stemness-related DEGs. We identified nine hub genes (AXL, EFEMP2, VIM, EHD2, COL3A1, FSTL3, ALOX5, TNFRSF12A, HSPB8) and defined them as stemness subtype classifiers by LASSO and RF machine learning methods. The AXL protein belongs to the TAM (TYRO3, AXL, and MER) family of receptor tyrosine kinases, which is an essential factor for stemness. Upregulation of AXL expression is correlated with resistance to TKIs and chemotherapeutic agents in various types of cancer, including LUSC [46]. EFEMP2 and EHD2 have been reported to inhibit the invasion and metastasis of lung cancer cells by regulating the epithelial-mesenchymal transition (EMT) process and MMP activity [47, 48]. HSPB8 is a stress-related protein that plays an important role in tumor proliferation, invasion and apoptosis in lung cancer [49]. FSTL3, as an oncogene of the FSTL family, is involved in the occurrence and progression of lung cancer [50]. Previous reports have demonstrated that FSTL3 is linked to remodeling of the tumor immune microenvironment and may serve as a predictor of sensitivity to immunotherapy and chemotherapy [51]. COL3A1 is an integral ECM protein that is closely involved in malignant progression and drug resistance by regulating tumor immunity and EMT in a variety of cancers, particularly lung cancer [52]. ALOX5 encodes a nonheme iron-containing dioxygenase of the lipoxygenase gene family that has been identified as a critical regulator of cancer stem cells from hematological malignancies [53]. TNFRSF12A is a member of the TNF superfamily of receptors that has been reported to be elevated in different cancers [54]. At present, the role and clinical value of these two genes in LUSC remain unclear. However, inhibition of these stemness-related hub genes may be a promising approach to gain a better therapeutic effect in LUSC patients.

In recent years, studies have confirmed that cancer stemness and stemness-related genes could serve as diagnostic, prognostic and therapeutic biomarkers in various cancers [55, 56]. Our classifier could identify two distinct stemness subtypes in LUSC patients and provide a possible method for screening LUSC patients who display an effective response to different treatment strategies. However, there are still several limitations in this study. The major limitation of this study was that all of these results were based on bioinformatics analysis of public databases. Although three GEO datasets were enrolled as an external validation set to verify the predictive efficiency and support the conclusions of our study, a clinical cohort from our own center to confirm the classifier is necessary. Furthermore, additional in vivo or in vitro experiments, such as flow cytometry or preclinical models, are warranted to comprehensively analyze the molecular mechanisms and verify our results.

## Conclusions

In summary, our study performed an in-depth analysis of the association between cancer stemness and the prognosis, immune infiltration status, and treatment response of LUSC patients. In this study, LUSC patients were divided into two stemness subtypes with distinct survival outcomes and immune infiltration statuses. We further developed a clinically applicable stemness subtype classifier that could conveniently discriminate the stemness subtype of LUSC patients. The classifier could assist physicians in predicting the patients’ prognosis and treatment response, and selecting effective treatment strategies for patients with LUSC.

## Electronic supplementary material

Below is the link to the electronic supplementary material.

Supplementary Material 1

Supplementary Material 2

## Data Availability

The TCGA and GSE30219, GSE37745 and GSE73403 datasets analyzed for our study can be found in the TCGA database (https://portal.gdc.cancer.gov/) and the GEO database (https://www.ncbi.nlm.nih.gov/geo/).
