# Supplementary material for: Characterization of stemness features and construction of a stemness subtype classifier to predict survival and treatment responses in lung squamous cell carcinoma
Source: BMC Cancer. 2023 Jun 8;23:525. doi: 10.1186/s12885-023-10918-y (PMC10251713; doi:10.1186/s12885-023-10918-y)
Supplement: Supplementary file 2 — Supplementary Material 2 [file 12885_2023_10918_MOESM2_ESM.docx]

**Figure S1**


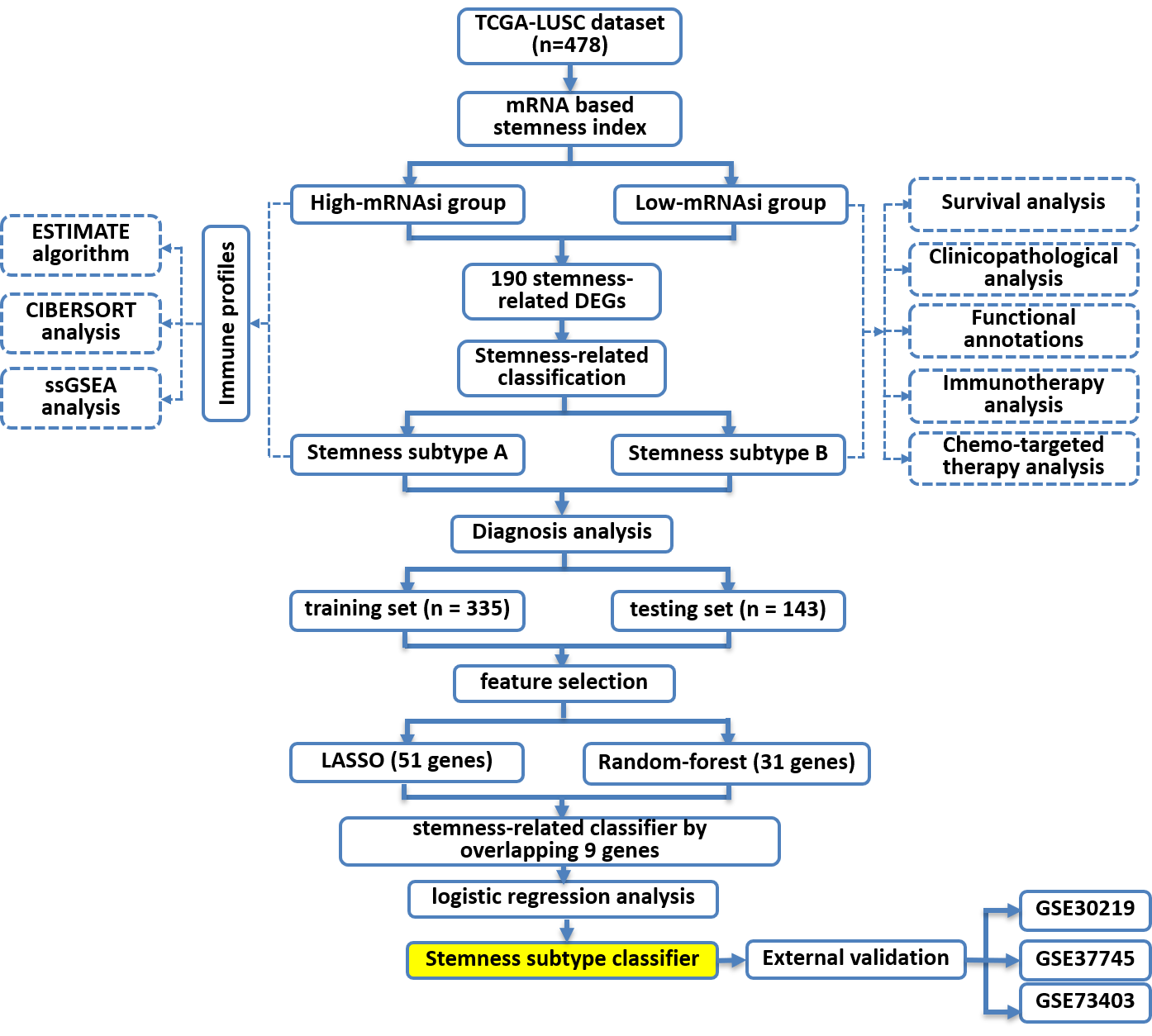


**Figure S1**: The workflow of our study

**Figure S2**

**
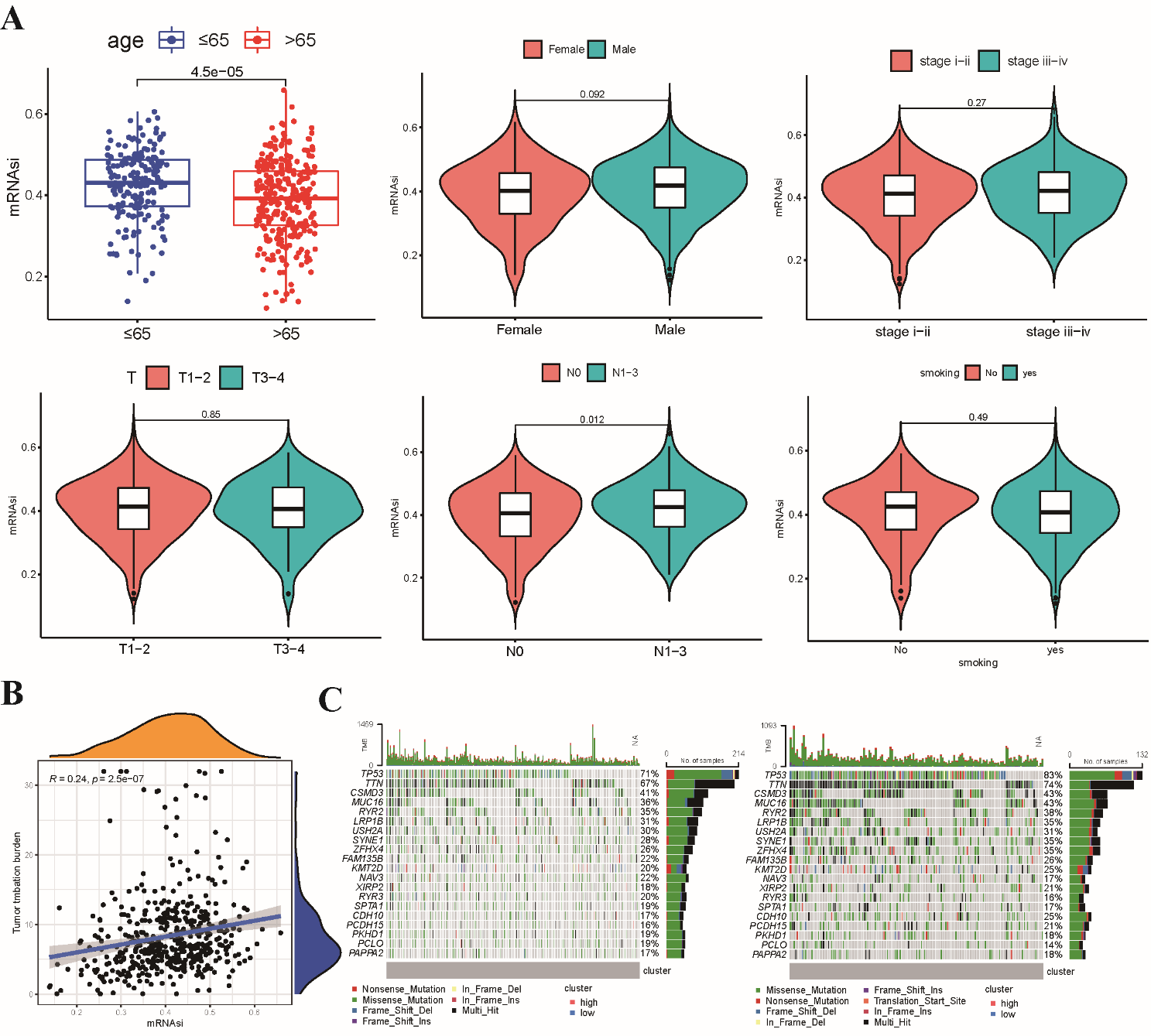
**

**Figure S2 (A)**The scatter plot showed that the mRNAsi scores among patients of different age, gender, clinical stage , T stage, N stage and smoking in LUSC. **(B)** There is a positive correlation existed between mRNAsi scores and TMB in LUSC patients. **(B)** The waterfall plots displayed the frequent 20 mutated genes in these two groups.

**Figure S3**


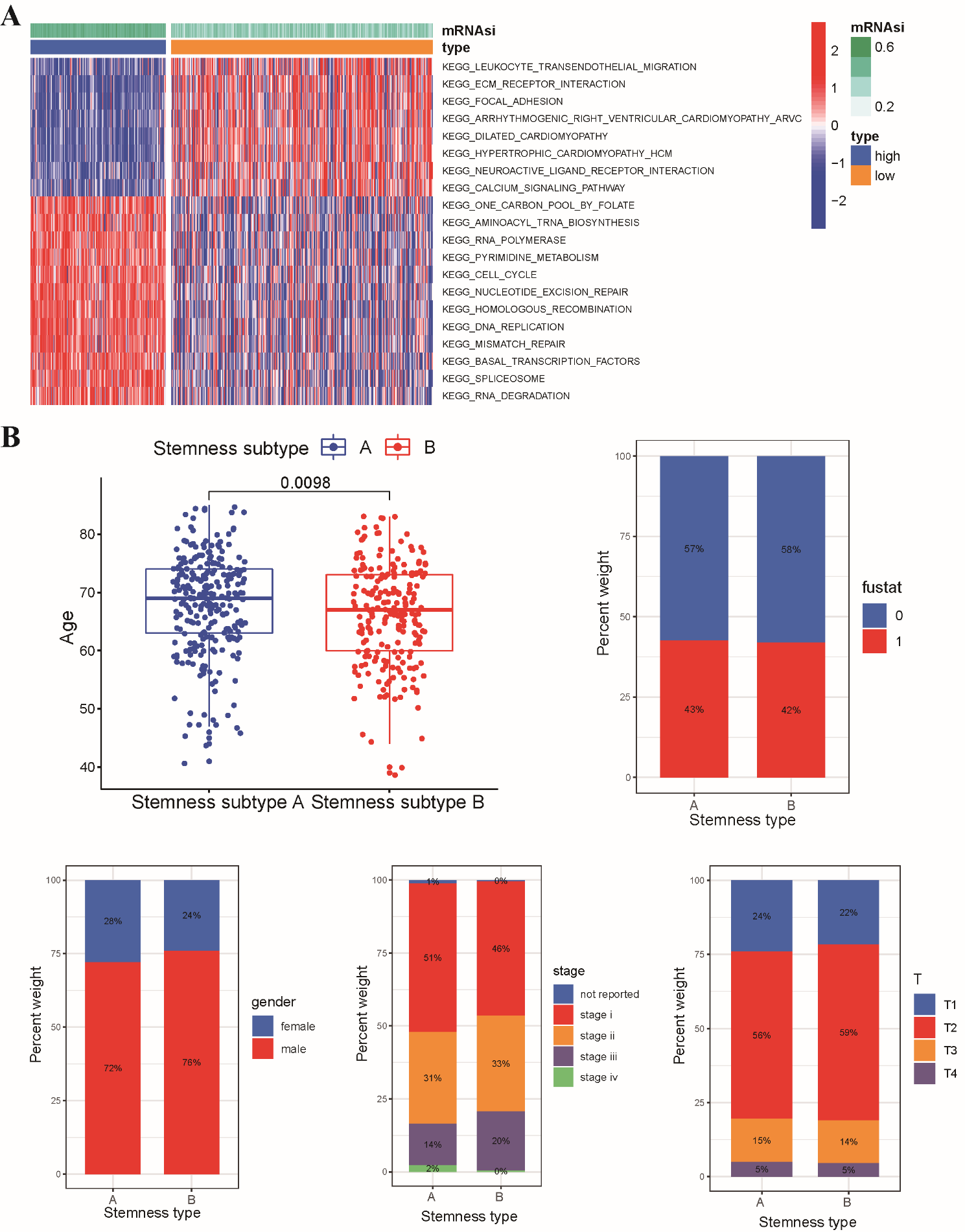


**Figure S3:** **(A)** Heatmap of GSVA analysis demonstrated the top 20 significantly enriched molecular pathways between low-mRNAsi and high-mRNAsi groups. **(B)**Stacked histogram showing the proportions of age, survival status, gender, T stage and clinical stage between stemness subtype A and B groups.

**Figure S4**


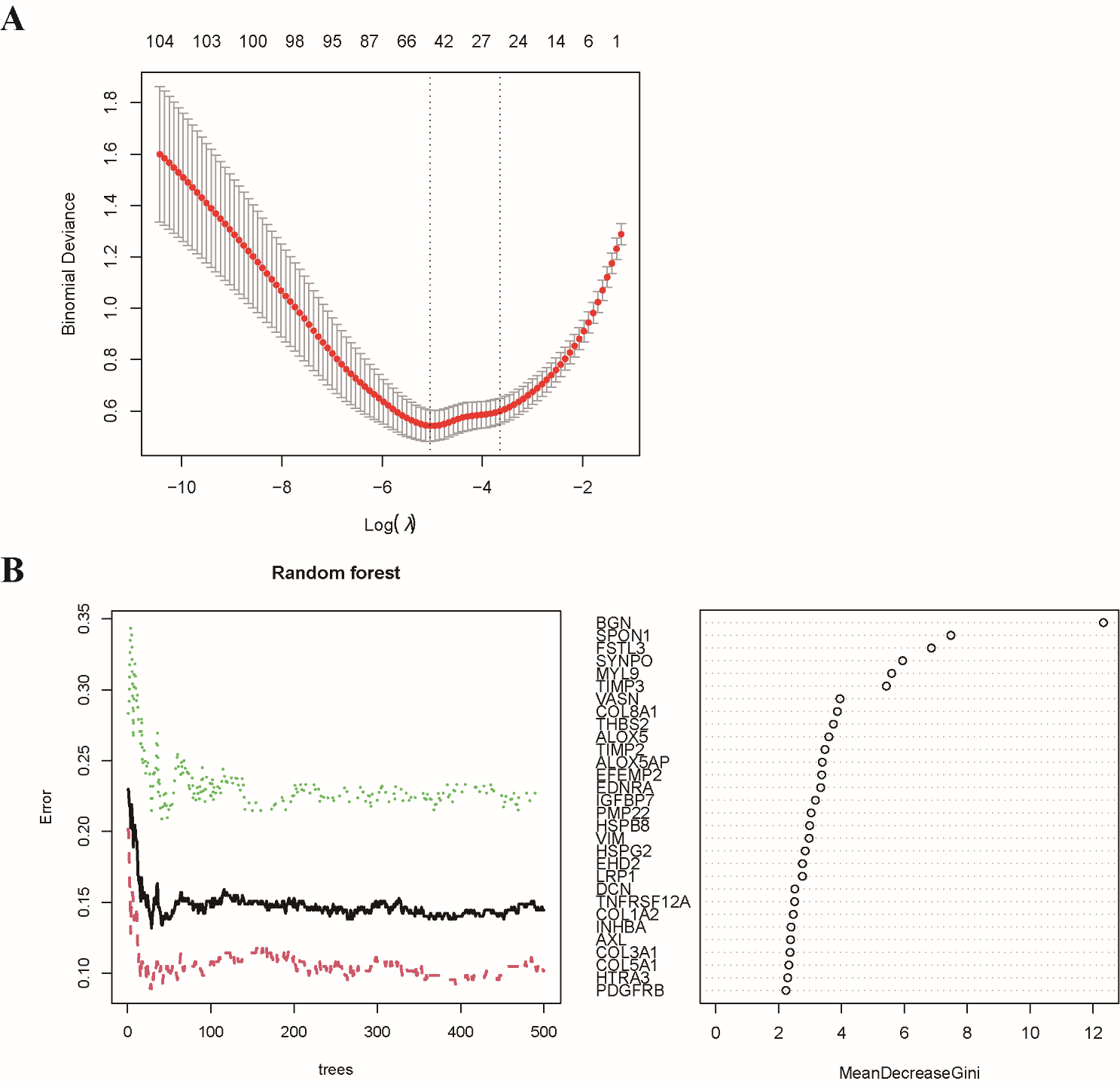


**Figure S4:** LASSO **(A)** and RF **(B)** analyses were applied to select the most hub genes associated with the stemness subtypes based on the expression levels of 190 stemness-related DEGs

**TABLE S1 |** Sequences of the primer used for qRT-PCR

| mRNA | Forward primer | Reverse primer |
| --- | --- | --- |
| COL3A1 | CTTCTCTCCAGCCGAGCTTC | TGTGTTTCGTGCAACCATCC |
| EFEMP2 | AAGAGCCCGACAGCTACAC | AGGGATGGTCAGACACTCGTT |
| EHD2 | TGGTGCGAGTTCACGCTTAC | ATGACGGGCAGTTTGAGGAT |
| ALOX5 | CCAAGTCGGTCAAGAGCC | TACATGCCCAGGAACAGC |
| FSTL3 | GTGCCTCCGGCAACATTGA | GCACGAATCTTTGCAGGGA |
| GAPDH | CCAACTGCCAGACTACCAC | GGACCAGGCTGTTCCAAGA |
